# Hyperglycemia diverts dividing stem cells to pathological adipogenesis

**DOI:** 10.1186/scrt518

**Published:** 2014-11-17

**Authors:** Vincent Hascall, Aimin Wang

**Affiliations:** Biomedical Research, Lerner Research Institute, Cleveland Clinic, Cleveland, OH 44195 USA

## Abstract

This commentary proposes a mechanism for why murine diabetic adipose tissue contains very few remaining stem cells compared with normal adipose tissue. The mechanism involves the diversion of stem cells to pathological adipocytes when they divide in hyperglycemia.

The interesting and important study by Rennert and colleagues [1], 'Diabetes impairs the angiogenic potential of adipose derived stem cells by selectively depleting cellular subpopulations', investigated properties of stem cells isolated from adipose tissue from normal and diabetic type 1 and type 2 obese mice. Their data show that adipose tissue from both types of diabetes contain significantly fewer stem cells that normally can form osteogenic and adipogenic progeny as well as a greatly decreased selective subpopulation that can participate in angiogenic processes such as wound healing. The authors conclude that 'these data suggest that the utility of autologous adipose tissue cells for cell-based therapies in diabetic patients may be limited'. As adipose tissue is now considered a very useful source of autologous stem cells for various translational therapies, this study provides strong evidence that adipose tissue from diabetics will have far fewer stem cells.

Our studies and others provide clues for the mechanisms involved. We have now shown that rat bone marrow stromal (stem) cells stimulated to divide in osteogenic medium in which the glucose concentration was increased from normal (5 mM) to hyperglycemic (25 mM) diverted to a pathological adipogenesis [2]. The mechanism involves activation of hyaluronan synthases (HASs) in intracellular membrane compartments. The HASs utilize cytosolic substrates, UDP-glucuronate and UDP-N-acetylglucosamine, and synthesis of hyaluronan is an effective way to lower influxed cytosolic glucose stress [3]. The HAS enzymes normally migrate to the plasma membrane before activation, and the elongating, polyanionic, very large hyaluronan macromolecules are extruded into the extracellular pericellular matrix. Activation of HASs in intracellular membrane compartments extrudes the hyaluronan macromolecule into the endoplasmic reticulum (ER), transport vesicles, and the Golgi. This initiates a unique autophagic ER stress mechanism that extrudes the hyaluronan into a monocyte-adhesive extracellular matrix after completing cell division. This mechanism also occurs with stromal/stem cells in trabecular bone of type 1 diabetic rats with subsequent osteopenia [2]. This provides evidence that a stem cell that divides in hyperglycemia produces two daughter pathological adipocytes with loss of a stem cell that would normally occur if the division were asymmetric.

The study by Chang and colleagues [4] showed that sections of adipose tissue from the obese mice, but not from normal mice, had extensive extracellular hyaluronan matrices around the adipocytes. The matrix was embedded with Mac-2 macrophages that likely were recruited to remove the hyaluronan matrix, which would otherwise accumulate indefinitely as the adipocytes would continue to synthesize and extrude hyaluronan in response to sustained hyperglycemia. The ability of monocytes/macrophages to remove the monocyte-adhesive matrix has been shown as described in the review [5]. This mechanism also appears to be active in kidney mesangial cells in type 1 diabetic rats that were treated daily with a small intraperitoneal dose of heparin, which sustains kidney function in this model [6].

Our proposed model, then, is that stem cells that divide in hyperglycemia (>2.5 times normal) divert to a pathological adipogenesis in response to the glucose stress, and that subsequent cell divisions along this pathway could contribute to the extensive population of fat cells in adipose tissue in diabetes. Further, this mechanism is occurring in other tissues that contribute to diabetic pathologies, including osteopenia, impaired wound healing, aberrant vascularization in necrotic feet, and so on. As indicated recently in an interesting article in the magazine *Time*, 'Don’t blame fat' [7], it is the high carbohydrate, low fat Western diet that is greatly contributing to the ongoing epidemic of diabetes and obesity.

## Abbreviations

ER: endoplasmic reticulum
HAS: hyaluronan synthase.
